# Editorial: Development and Applications of New Activity-Based Probes

**DOI:** 10.3389/fchem.2021.754294

**Published:** 2021-09-09

**Authors:** Galia Blum, Steven H. L. Verhelst, Xiaowei Ma

**Affiliations:** ^1^ The Faculty of Medicine, The School of Pharmacy, The Institute for Drug Research, The Hebrew University of Jerusalem, Jerusalem, Israel; ^2^ The Wohl Institute for Translational Medicine, The Goldyne Savad Institute for Gene Therapy, Hadassah Hebrew University Medical Center, Jerusalem, Israel; ^3^ KU Leuven, Department of Cellular and Molecular Medicine, Leuven, Belgium; ^4^ Leibniz Institute for Analytical Sciences ISAS, Dortmund, Germany; ^5^ Department of Nuclear Medicine, The Second Xiangya Hospital, Central South University, Changsha, China

**Keywords:** protease probes, activity-based probe, recognition element, ABP generation, ABP application

Tools that detect the activity of enzymes can be utilized in several ways: for studying enzyme involvement in both healthy and pathological conditions, for imaging applications, as diagnostic reagents and for screening of new drug leads. Activity-based probes (ABPs) are a class of chemical tools that bind to active enzymes and report on their activity. In contrast to substrate reporters, ABPs covalently attach to their active enzyme target and covalently link a reporter to it. The covalent modification enables studying the fraction of active enzyme in systems such as purified enzymes, cell lysates, intact cells and even whole animals. The enzyme–ABP complex can be detected using several methods including microscopy, fluorescent activated cell sorting (FACS), gel analysis, *in vivo* imaging, or by mass spectrometry.

Although the above applications are highly informative and extremely useful, they are usually applied in a low throughput manner. In this special issue of Frontiers, Jones et al. have elegantly described methods for high-throughput ABPP (ABPP-HT) using a pan deubiquitylating enzyme (DUB) targeted probe for proof of concept. Their methodology, which combines short chromatography gradients and fast scan speeds (using Parallel Accumulation Serial Fragmentation (PASEF) data acquisition), allows increased sample throughput in a microplate format and rapid analysis of up to 300 samples per day. The authors were able to efficiently analyze a few inhibitors on a panel of 30–40 DUBs in two cell lines and mouse brain lysate. These novel methodologies bring the use of ABPs a big leap forward in the attempt to study the activity of proteases in a complex proteome.

One of the biggest challenges in the field of ABPs is developing selective probes that detect a single enzyme. The underlying reason is close substrate specificity between related enzymes. Selective ABPs are difficult to generate, even using positional scanning libraries. Recently, new approaches have been brought to the protease field, such as hybrid combinatorial substrate libraries (HyCoSuL) that uses unnatural amino acids in a substrate library (Poreba et al., 2014) and phage display (Chen et al., 2021). Nevertheless, pan-selective probes that label a subset of enzymes have their own advantages: they usually produce stronger signals than selective probes, thus enabling easier detection. In addition, the activity of specific enzymes can be analyzed biochemically, for example by gel-based methods, enabling the monitoring of differences in activity of a group of enzymes at once. The application of selective probes in comparison with a pan-protease probes is nicely demonstrated in the work of van Dalen et al. who generated a selective cathepsin S ABP with help of docking experiments. After probe generation and evaluation, it was applied to study cathepsin S activity in bone marrow derived macrophage cells treated with *E. Coli*. Images were generated using confocal and correlative light-electron microscopy imaging showing vesicles that contain active cathepsin S but no other cathepsins.

While most ABPs include recognition-elements derived from peptides/proteins (including natural or non-natural amino acids), this issue of Frontiers includes two manuscripts describing ABPs based on inhibitors (Van Rymenant et al.); (Keuler et al.). Van Rymenant et al. describe probes selective towards Fibroblast activation protein (FAP) and Keuler et al. describe their success in generating inhibitor based ABPs targeted to NLRP3. The FAP probes were evaluated on recombinant enzymes and cell lysates, and they were useful in monitoring FAP activity in transfected intact cells as well as in urothelial cancer tissue sections (Van Rymenant et al.). The NLRP3 probes were evaluated for their target binding, influence on bone marrow-derived macrophages viability, cytokine secretion and ability to detect NLRP3 in cells by microscopy (Keuler et al.). The activity of both these important targets can now be studied using ABP methodology.

An interesting review by Van der Zouwen et al. nicely describes several methodologies for generating libraries of ABPs, opposed to conventional synthetic methods that generate individual probes one at a time. Modular probe synthesis and probe modifications were described including DNA-encoded libraries, phage display, multicomponent reactions (MCR), Sulfur (VI) ﬂuoride exchange (SuFEx) click chemistry, cyclization and trifunctional reagents, all to generate versatile probes. The advancements in probe generation indeed open ample opportunities for directing novel probes to new targets using novel applications.

A review by Burster et al. describes the development and use of a variety of ABPs including quenched probes, CyTOF labeled probes, fluorescent and radioactive probes. They then focus on cathepsin G probes and describe their application in studying intracellular and extracellular activity. In addition, a review by Kasperkiewicz methodically goes through the components of ABPs and discusses each in depth, the history, advantages and disadvantages and their incorporation within serine ABPs. A nice comparison is made between substrates probes and ABPs describing their use and limitations. The generation and choice of tag, recognition element and electrophile are discussed as well as the application of serine ABPs to various systems. The Kasperkiewicz review is a thorough overview on serine ABPs that share ample details on specific probes targeted to serine proteases, with a focus on human neutrophil elastase, cathepsin G and Kallikreins.

In conclusion, this special edition of Frontiers in Chemistry Chemical Biology gives a broad and in-depth- overview on ABPs, their generation and applications. It also describes various new ABPs and novel applications for a variety of proteases.
